# Effect of Gear Pump Extrusion Processing on the Properties of Fiber Reinforced Rubber Composites

**DOI:** 10.3390/polym12040985

**Published:** 2020-04-23

**Authors:** Xiaolong Tian, Lin Zhu, Kunling Li, Kongshuo Wang, Huiguang Bian, Lin Li, Shaoming Li, Chuansheng Wang

**Affiliations:** College of Electromechanical Engineering, Qingdao University of Science and Technology, Qingdao 266061, China or 15165268516@163.com (X.T.); qustzhulin@163.com (L.Z.); likunling1208@sina.cn (K.L.); kongshuo726@163.com (K.W.); bianhuiguang@163.com (H.B.); ll@qust.edu.cn (L.L.); lishaoming@qust.edu.cn (S.L.)

**Keywords:** short fiber reinforced rubber composites, processing technology, gear pump auxiliary extrusion, composite properties

## Abstract

Short fiber reinforced rubber composites have been widely used in rubber products attributed to their excellent wear resistance. However, there are still some serious problems in the processing of short fiber reinforced rubber composites, such as Mooney viscosity increasing, temperature and extrusion pressure rising, and unstable extrusion quality. In particular, short fibers need to be extruded in a specific direction during the molding process, and the problems in this process are particularly prominent. In this manuscript, the influence of gear pump assisted extrusion on the properties of short fiber rubber composites is studied. The experimental results show that the application of a gear pump to assist extrusion could significantly increase the die pressure, reduce the extrusion temperature of the blend, and improve the extrusion efficiency and stability of the blend. Furthermore, it could improve the vulcanization efficiency, increase the tensile strength and tear strength of the compound, reduce wear, and guarantee the quality of extrusion products.

## 1. Introduction

Short fiber has been widely used to reinforce rubber composites of rubber products, such as tires, conveyor belts and seal [1,2,3,4]. The short fiber addition could generally improve the comprehensive performance of rubber composite, such as hardness, modulus, corrosion resistance, compressibility, and wear resistance. To achieve the best performance of those rubber products, some factors, such as sufficient interaction between fiber and the matrix, sufficient dispersion of fibers, high aspect ratio during processing, and desired orientation in the desired direction [5], have to be carefully considered [1].

Because the molecular structure, reactivity, and surface properties of the short fiber and rubber matrix are different, the adhesion between them [6,7,8] is difficult and it has been the primary factor in the preparation of short fiber reinforced rubber composite [9,10]. Many methods were found to improve the interaction between fiber and rubber for these differences. The bonding reaction between the rubber and the fiber was mainly carried out by a chemical method, thereby enhancing the adhesion between the short fibers and the rubber molecules [11]. This is a common method to enhance the adhesion behavior between rubber and short fiber interface by chemical modification [12]. These methods are very effective and have been widely used. 

**Figure 1:**
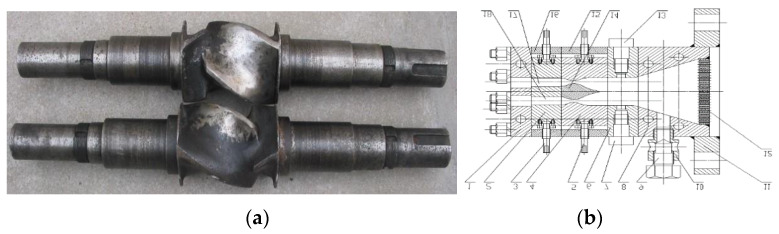
Equipment for short fiber processing and orientation: (**a**) Rotor of tangential types internal mixer for short fiber mixing; (**b**) Structure of dam-expansion die for short fiber radial orientation.

The processing of short fiber reinforced rubber composite is significantly important, which may receive a better dispersion of fibers, a high aspect ratio during processing and controlling the orientation in the desired direction for application [13]. There have been many researches on the processing technology of short fiber rubber composite [14,15,16,17,18]. Hintze et al. [1] studied the influence of different processing conditions and fiber contents on the resulting morphology and macroscopic properties. Lekube et al. [19] investigated the influence of processing temperature and screw configuration in compounding on the properties of glass fiber reinforced polypropylene. In previous work [20,21,22], the mixing methods of short fiber rubber composite and extrusion process with a radial orientation of short fiber in a rubber matrix have been researched. Some equipment and the process technology have been produced, as shown in Figure 1. However, there are some problems in the extrusion process of using short fiber radial orientation to reinforce rubber composites. The Mooney viscosity of those composite material increases [23], which causes many unfavorable factors in the extrusion process of the product, such as the increase of extrusion pressure, increase of extrusion temperature, and unstable extrusion quality. These phenomena are even more serious when short fibers orient in the radial direction during extrusion molding. 

In order to solve the above-mentioned problems, a gear pump has been applied in the extrusion molding process for short fiber reinforced rubber composite. The gear pump could convey positive displacement, to improve the extrusion stability of the product. Meanwhile, the pressure building function of the extruder can be transferred to the gear pump to reduce the temperature of the rubber composite during extrusion and thus the pumping capacity of the extruder equivalent is improved. In this paper, the melt gear pump auxiliary device was used in conjunction with the extruder to study the processing difficulty and performance of the short fiber reinforced rubber composite at different speeds. 

## 2. Materials and Methods 

### 2.1. Materials

Table 1 shows the different components of the rubber composite formulation. Natural Rubber (NR) SVR3L was bought from Dakruko, Buon Ma Thuot, Vietnam. Carbon blacks N330 was obtained from Jiangxi Black Cat Carbon Black Co., Ltd., Jiangxi, China. Silica HD 115 MP, was obtained from Rhodia White Carbon (Qingdao, China) Co., Ltd. ZnO was bought from Hebei Shijiazhuang Zinc Oxide Factory, Shijiazhuang, China. The oil types added to the NR compounds were VIVATEC500 naphthenic oil from Hansen & Rosenthal Chemical Group, Hamburg, Germany. Anti-aging agent 4020 (*N*-(1,3-dimethylbutyl)-*N0*-phenyl-*p*-phenylenediamine) and Accelerator NS were bought from Shandong shangshun Chemical Co., Ltd., Weifang, China. Stearic acid was bought from Fengyi Grease Technology (Tianjin, China) Co., Ltd. Sulphur was bought from Chaoyang Tianming Industry and Trade Co., Ltd., Beijing, China. Coupling agent Si-69 was supplied by Shandong Ningjin haorun Technology Co., Ltd., Dezhou, China. Resin SL3020(1,3-Dihydroxybenzene), was supplied by Sino Legend (China) Chemical Company Ltd., Suzhou, China. Adhesive RA65, was supplied by Wuxi Huasheng Rubber New Material Technology Co., Ltd., Wuxi, China. Aramid short fibers were supplied by Baoding New Urban Synthetic Material Factory (Baoding, China), with an average initial length of 3 mm and fiber diameter of 10–12 microns.

### 2.2. Adhesion Mechanism of Short Fiber to Rubber Matrix

**Figure 2:**
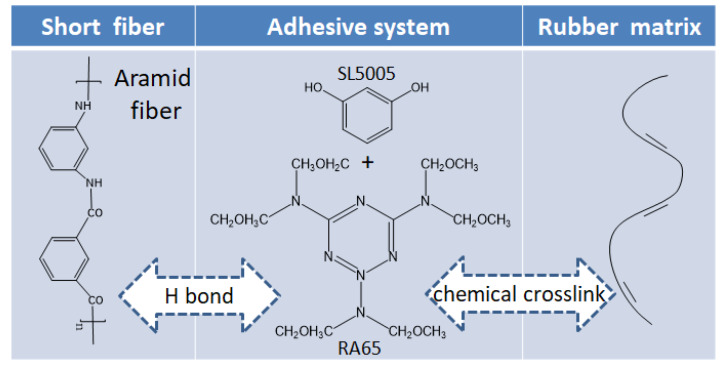
Mechanism of adhesion system.

Adhesion between short fiber and rubber matrix is the key to ensure the properties of composite materials. The adhesive system, as an active intermediate, participates in the adhesion process between short fiber and rubber matrix [24,25]. The specific adhesion mechanism is shown in Figure 2.

**Figure 3:**
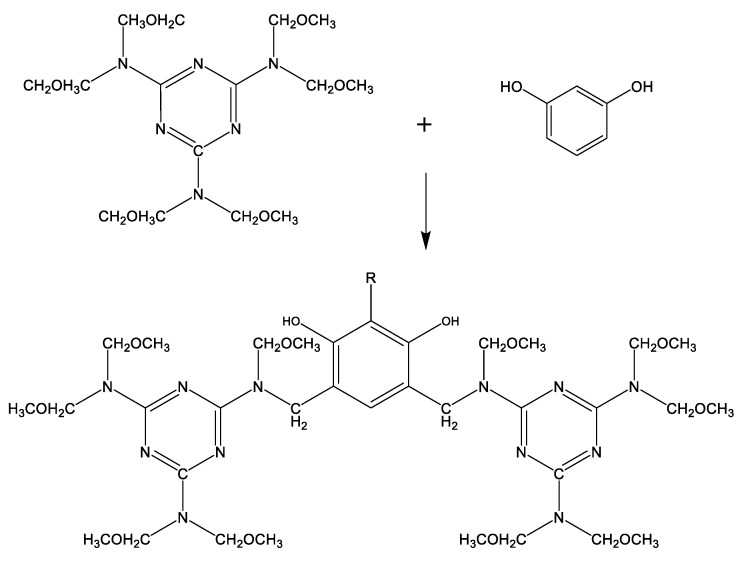
Formation process of active intermediates.

**Figure 4:**
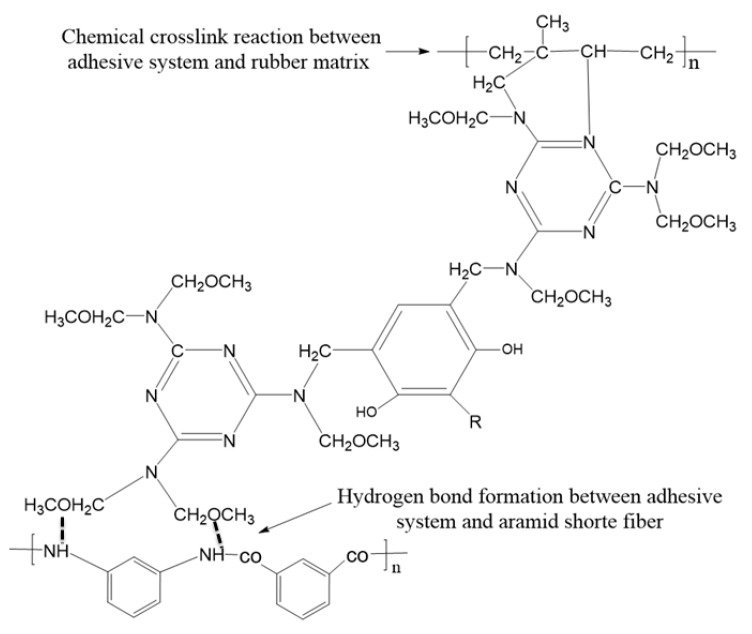
Adhesion process between short fiber and rubber matrix.

The adhesive system is uniformly dispersed in the rubber matrix after mixing, the chemical components in the adhesive system react first to form active substances in the process of vulcanization, which can react with rubber and short fiber. Figure 3 describes the chemical reaction process. The active intermediate substances then undergo two important reactions, the first reaction is to form a chemical crosslink with rubber matrix, and the other reaction is to form a hydrogen bond with the short fiber, so as to realize the bonding between short fiber and rubber matrix. Figure 4 depicts the entire adhesion process.

### 2.3. Processing Methods

The processing of rubber composites mainly consists of three steps. The first step is the mixing process of rubber composites, which mainly aims to realize the uniform dispersion of materials such as short fibers in the rubber matrix. The second step is the extrusion of rubber composites. In this step, the properties of short fiber rubber composites are investigated in the process of gear pump assisted extrusion. This step is the main content of this study. The third step is the vulcanization of composite materials.

#### 2.3.1. Mixing Process

The mixing process of short fiber rubber composite includes a two-step mixing process of internal mixing and open mixer mixing. In the first stage, the fiber, rubber and most materials were incorporated on a laboratory internal mixing X(S)M-1.7L. In the second stage, Sulphur (resin, and adhesive) was added into the two-roll mill XK-160. The mixing parameters and mixing processing were listed in Table 2.

#### 2.3.2. Extrusion Process

**Figure 5:**
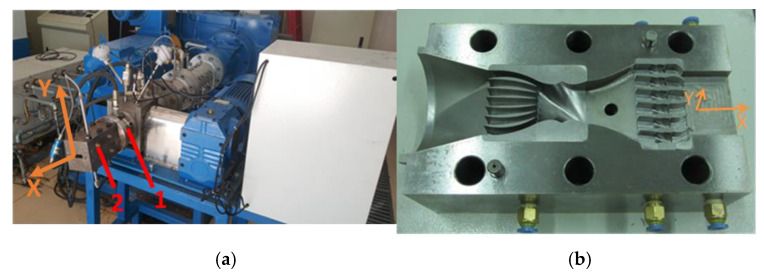
(**a**) Short-fiber radial orientation direction: 1 is referred to Herringbone gear pump, 2 is referred to orientation die; (**b**) Structure of orientation die.

In order to study the effect of gear pump processing on the properties of short fiber reinforced rubber composites, Herringbone gear pump (Hatec, Germany) and orientation die (manufactured by Qingdao University of Science and Technology, Figure 5b) were installed on the extruder in sequence. The general orientation of the fiber extrusion process was along the extrusion direction (x-direction), orientation die could implement the short fiber radial orientation(y-direction). Figure 5 shows the short-fiber radial orientation direction and the detailed structure of orientation equipment.

The mixing rubber was added into extruder XJD-65, manufactured by Inner Mongolia Ford Rubber Machinery Company. The extruder cooling water temperature is 70 °C. Gear pump cooling water temperature is 40 °C. Gear pump speed is 25 rpm. Table 3 shows the extrusion experimental design.

#### 2.3.3. Vulcanization Process

**Figure 6:**
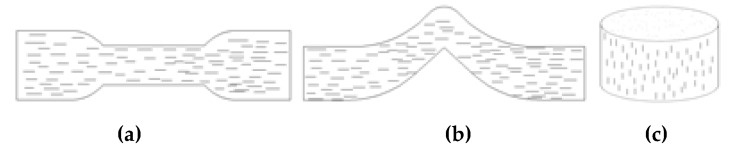
The testing specimen: (**a**) Sample for tensile; (**b**) Sample for tear; **(c**) Sample for wear.

The orientation direction must be considered during the vulcanization process. The testing specimen is the fiber orientation direction, as illustrated in Figure 6. The rubber compounds were cured at 150 °C at a pressure of 10 MPa for optimum cure time (t90) + 3 min.

### 2.4. Characterization

Extrusion stability: The cutter is installed in front of the extruder head, and the cutter speed is 6 rpm. When the rubber composites were continuously extruded from the extruder, the cutter was started and the running time was 3 min. The collected rubber compound was weighed to test the extrusion stability of the rubber compound.

Degree of compaction: The degree of compaction of the rubber compound after extrusion was observed using an optical microscope (LEXT OLS4000, OLYMPUS). The specimen was sliced in the extrusion direction after cooling the extruded rubber mixture to room temperature.

Curing Characterization: Curing characterization of rubber compounds were evaluated using a moving-die rheometer (M-2000-AN) from GOTECH TESTING MACHINES CO., Ltd., Taichung City, Taiwan. The specimens were tested according to ISO 6502-2:2018. The Mooney viscosity values of the rubber compounds were evaluated using a Mooney viscometer (UM-2050, GOTECH TESTING MACHINES CO., Ltd.) according to ISO 289-2:2016.

Physical and Mechanical Properties. The hardness of the rubber vulcanizate was evaluated using Shore Hardmeter (LX-A, Shanghai Liuling Instrument Factory) according to ISO 7619-2: 2004, and three points were measured for each sample and the final result selected the median [6]. The tensile and tear properties of the vulcanized rubber were tested using a universal testing machine (TS 2005 b, GOTECH TESTING MACHINES CO., Ltd.) at a drawing rate of 500 mm/min according to the standards ISO 37: 2005 and ISO 34-1: 2004, respectively [6]. Five specimens were tested for each sample type and the final result selected the median. The abrasion of the rubber vulcanizates were evaluated using a DIN wear machine (GT-2012-D, GOTECH TESTING MACHINES CO., Ltd.) according to GB/T 1689-1998, and three specimens were tested and the final result selected the median.

Dynamic Mechanical Thermal Analysis: The viscoelastic mechanical properties of the vulcanizates were evaluated using dynamic thermomechanical analysis (EPLEXOR-150N, Gabo Qualimeter Testanlagen GmbH). The test temperature range is from −65 °C to 65 °C in tensile mode at a heating rate of 2K per minute. The frequency is 10 Hz. The static strain is 5%. The static force is 70 N. The dynamic strain is 0.25%. The dynamic stress is 60 N.

Morphology Analysis. The cross section of the sample after tensile fracture was observed under a scanning electron microscope (JSM-7500F, Japan Electronics Corporation).

## 3. Results and Discussion

### 3.1. The Pressure and Temperature of the Rubber Compound during Extrusion

Extrusion temperature and pressure change are important indicators for evaluating the forming process of short fiber rubber composites. Table 4 shows the data of different extrusion processes.

It can be seen from Table 4 that, at the same screw speed, the relationships of maximum pressure and extrusion temperature of the mixing rubber at the extrusion head is Y-1 > X-1, Y-2 > X-2, Y-3 > X-3. With the increase of screw speed, compared with X-1, the pressure of the extrusion head of X-3 increased by 1.74 MPa, and the extrusion temperature increased by 15 °C. Compared with Y-1, the pressure of the extrusion head of Y-3 increased 2.26 MPa, and the extrusion temperature increased 18 °C. It is indicated that short fiber rubber composite has a higher viscosity and it is difficult to be extruded. This is because the short fiber has a higher modulus, which greatly increases the effective volume, stress and strain of the filler. The extruder needs to provide more pressure to ensure material handling, which makes the heating rate of the mixing rubber to accelerate. As the screw speed increases, the shear rate of the material in the groove increases, and the temperature rise is further increased, which may cause rubber burning and affect the performance of the rubber compound.

The gear pump was used in the process of the extrusion. The relationships of maximum pressure and maximum temperature in the extruder are Y-1 > Z-1, Y-2 > Z-2, Y-3 > Z-3. This is because the gear pump provides the pressure-building function, making the internal pressure of the extruder reduced and the temperature rise decreased. At the same screw speed, the extrusion temperature relationships of rubber compound are Y-1 > Z-1, Y-2 > Z-2, and Y-3 > Z-3, while the extrusion head pressure relationships are Z-1 > Y-1, Z-2 > Y-2, and Z-3 > Y-3. As the screw speed increases, the pressure of the head of Z-3 increased 3.32 MPa compared with the Z-1 and the extrusion temperature of the rubber compound increased only 15 °C. The results indicated that the gear pump assisted extrusion process could increase the extrusion head pressure and reduces the extrusion temperature of the rubber compound. This is because the flow path of the rubber compound in the used gear pump is short, and the generated shear heat is low, while the range of temperature rise is small. With the increase of the screw speed, the temperature increase is not large, which is beneficial to maintain the performance of the extrusion rubber at high speed.

### 3.2. Extrusion Stability

**Figure 7:**
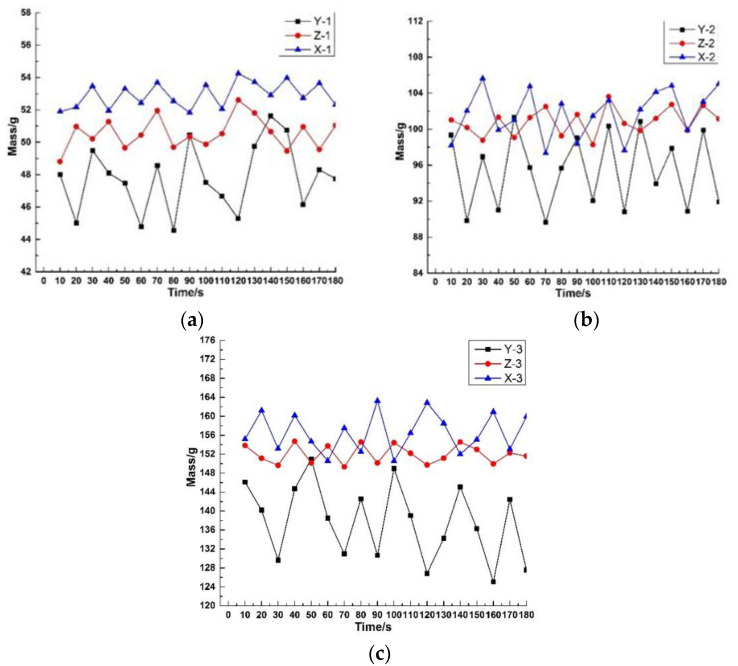
Weight change of rubber block under different extrusion processes: (**a**) The weight of the extruded block when the extruder speed is 10 rpm; (**b**) The weight of the extruded block when the extruder speed is 20 rpm; (**c**) The weight of the extruded block when the extruder speed is 30 rpm.

In the process of continuous and stable extrusion, the extruded rubber was cut and weighed in a certain time interval. The weight change of the extruded rubber compound under different extrusion process conditions is shown in Figure 7.

In the same extruder speed, the average weight relationships of the extruded rubber compound is X-1 > Z-1 > Y-1, X-2 > Z-2 > Y-2, and X-3 > Z-3 > Y-3. This is because aramid short fibers mixed in the rubber matrix increased the viscosity of the rubber and the pressure of the die, which caused the pressure reflux and leakage flow in the extruder to increase, and the extrusion volume to decrease. When the gear pump is used to assist extrusion, the two intermeshing gears feed the compound into a confined space and extrude the rubber mixture at the exit, so the pressure backflow and leakage flow of the head could be omitted. When the extruder screw speed is 30 rpm, the average weight ratio of Z-3 is increased by 10.95%, and the extrusion efficiency is significantly improved.

Extrusion stability generally decreases as the extruder screw speed increases. The standard deviation of the weight of X-3 extruded block increased from 0.77 to 4.05 than the X-1. The standard deviation of Y-3 from the weight of the Y-1 extruded block increased from 2.05 to 7.77. The standard deviation of the Z-3 to Z-1extruded block weight increased from 0.95 to 1.85, indicating that the extrusion stability of the short fiber matrix was lower than that of ordinary rubber compounds. When the gear pump is used to assist extrusion, the extrusion stability with short-fiber compound is improved. This is because the gear pump has the characteristics of positive displacement conveying, which can improve the pressure fluctuation during the extrusion of the rubber compound. Therefore, the gear pump assisted process can improve the extrusion efficiency of the short fiber mixture and ensure the extrusion stability.

### 3.3. Degree of Compaction

**Figure 8:**
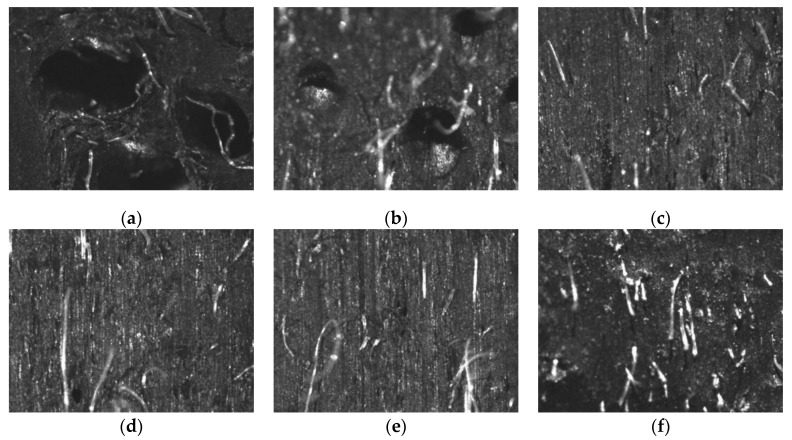
The compactness of rubber compound: (**a**) Y-1; (**b**) Y-2; (**c**) Y-3; (**d**) Z-1; (**e**) Z-2; (**f**) Z-3.

In the different process conditions, the compaction degree of the extruded short fiber rubber compound under the optical microscope is shown in Figure 8.

The pores are clearly visible in Figure 8a,b because the pressure at the extruder head is low at lower screw speed, and it is difficult to make extrusion molding when the Mooney viscosity of the short fiber rubber composite is large. At the same time, those factors reduced the density of the composite and the quality of the extruded product.

Pores inside the rubber compound are not obvious in Figure 8d–f. When the gear pump was used in the extrusion process, it could increase extrusion pressure, and keep the compound at a high pressure in the extruder head position. That could increase the degree of adhesion between the layers, thus ensuring the quality of the extruded product.

### 3.4. Mooney Viscosity and Vulcanization Characteristics

The Mooney viscosity and vulcanization characteristics are important indicators for evaluating polymer extrusion molding [26]. Table 5 shows the Mooney and vulcanization characteristics of the rubber compound under different extrusion process conditions.

As can be seen from Table 5, The Mooney viscosity will be significantly increased when the short fiber was added to the rubber compound. The Mooney viscosity decreases as the screw speed increases from the data of X-1, X-2, and X-3. The screw speed increased and leads to the increase of the shearing force and the number of shearing, making the dispersion of the filler become better and the Mooney viscosity lower. From the data of Y-1, Y-2, and Y-3, the Mooney viscosity of the rubber mixture appeared down at first and then rise. This is because the extrusion pressure of fluctuation is increased, when the screw speed increased. The dispersion of the short fibers is deteriorated, then the flow ability of the rubber mixture is lowered. When the gear pump was used in the process of extrusion, the Mooney viscosity gradually decreases with the increase of the screw speed, which indicates that the gear pump can ensure the dispersion of short fibers in the rubber compound at high speed extrusion.

In order to ensure the reinforcing durability of the short fibers in the orientation direction, the resin SL3020 and the adhesive RA65 were incorporated in the processing of the short fiber rubber composite to form the chemical bond between the short fibers and the rubber molecular in the vulcanization stage. These cross-linking structures keep the orientation of short fibers unchanged when the short fibers composite rubber is subjected to stress. However, this cross-linking reaction consumes a portion of zinc oxide [24,25], which weakens the concentration of the formulation activation system, thereby slowing down the vulcanization rate of the formulation. This is why the t90 of Y-1–Y-3 and Z-1–Z-3 is prolonged relative to X-1–X-3. At the same time, the cross-linking of short fiber and rubber molecular chain improves the cross-linking density of the total formulation system, so MH is increased and the tensile strength at a given elongation is increased.

### 3.5. Physical and Mechanical Properties

The physical and mechanical properties of the vulcanizate at different extrusion conditions are shown in Table 6.

As can be seen in Table 6, the hardness of the cured short fiber reinforced rubber composites is significantly increased by about 10% compared with that of the ordinary rubber composite. The short fibers added into the rubber matrix are involved in the crosslinking reaction, enhancing the effect of the reinforcement. The tensile and tear strength of the short-fiber vulcanizate is higher and the DIN wear value is lower than that of the normal vulcanizate. This indicates that the short fiber reinforced rubber composite performed a good anisotropy. 

The tensile and wear values of Y-3 were significantly reduced, while its tensile strength and DIN wear values are not as good as X-3. The screw rotation speed, the pressure of the extruder, and the extrusion pressure fluctuation is increased, the stability of the extrusion rubber compound decreases, the rubber material flow is disordered, and the physical and mechanical properties of the composite are degraded due to the agglomeration of the short fiber. 

The physical and mechanical properties of Z-1, Z-2, Z-3 are greater than that of Y-1, Y-2, Y-3, especially when the screw speed increases to 30 rpm, At this screw speed, the short fiber vulcanizate of Z-3 still maintains good performance, and it is even slightly improved. This is because the rubber compound is pressurized in the gear pump, and the pressure is further increased when passing through the extruder head. It is enhanced that shear tensile action of short fibers is in the flow path of the extruder head. The radial orientation of short fiber became higher, and its compaction increased. As the positive displacement conveying characteristics of the gear pump, it can be greatly reduced the influence of pressure fluctuation, improved the extrusion stability, and ensured the physical and mechanical properties of the extruded product.

### 3.6. Dynamic Thermo-Mechanical Analysis 

**Figure 9:**
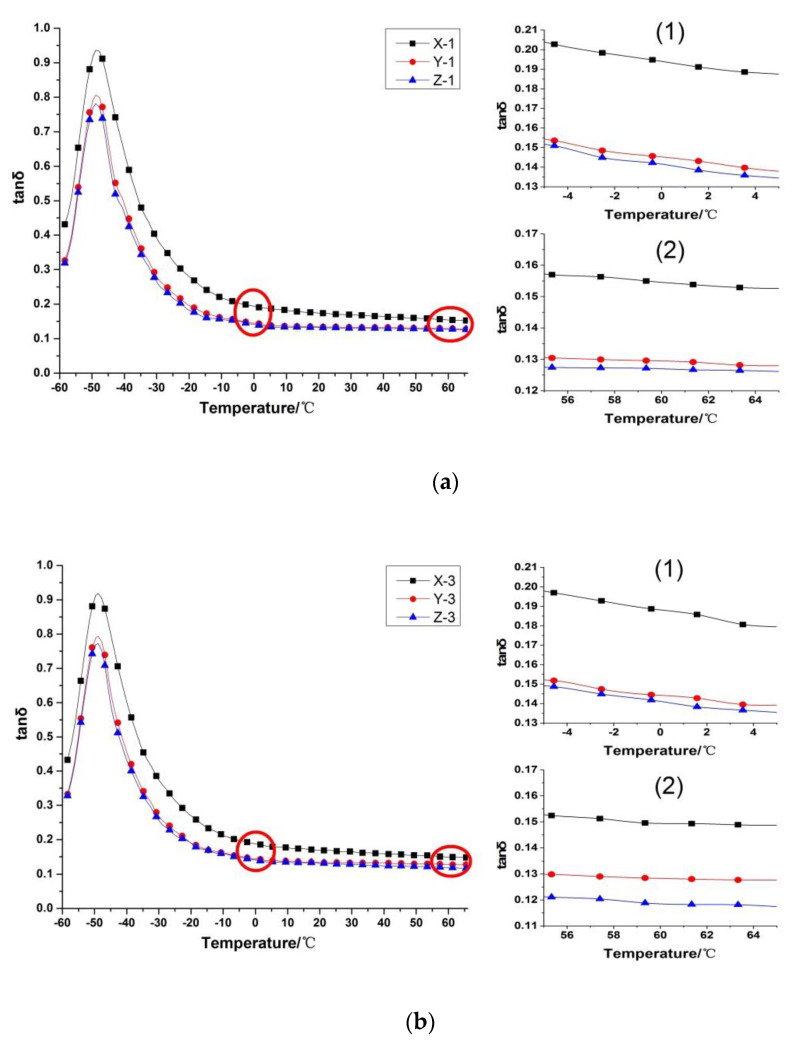
Tanδ-T Curve of vulcanizate: (**a**) Tanδ-T curve of vulcanizate when the screw speed is 10 rpm, 1 and 2 are magnified graphs of curves around 0 °C and 60 °C, respectively; (**b**) Tanδ-T curve of vulcanizate when the screw speed is 30 rpm, 1 and 2 are magnified graphs of curves around 0 °C and 60 °C, respectively.

The loss factor (tanδ) of the vulcanizate is an important indicator that can be used to characterize the dynamic mechanical properties of rubber composites [27]. Generally, tan δ at 0 °C is used to characterize the wet skid resistance of the composite [25,26]. The larger the tan δ value is, the better the wet skid resistance. The tan δ at 60 °C is used to characterize the rolling resistance of the composite [28,29]. The higher the tanδ value is, the higher the rolling resistance. The tanδ of the vulcanizate at 0 °C and 60 °C in different extrusion process conditions is shown in Figure 9.

The loss factor curve of vulcanized short fiber rubber composite is moved downward, and the loss factor (tanδ) is decreased. This shows that the heat build-up properties of the composites decreased, skidding behavior worse and rolling resistance lower when short fibers were incorporated into the rubber composites. Chemical cross-linking between short fibers and rubber molecular chains formed under the action of resin, so the rigidity of short fiber rubber composites are increased. Under the action of external force, the slippage of the rubber molecules is reduced, thereby reducing the slip friction between the rubber molecular chain and the filler particles, the heat generation is reduced, and the loss is reduced. 

The dynamic mechanical properties of short fiber rubber composites processed by gear pumps have also been improved. The difference value of loss factors between Z and Y increases at 0 °C and 60 °C. These indicate that the gear pump used in the extrusion process of the short fiber rubber composite material, could improve the dynamic mechanical properties of the composite material, enhance the wet-slip resistance of rubber composites and reduce the rolling resistance of materials.

### 3.7. Scanning Electron Microscopy (SEM) Observation and Analysis

**Figure 10:**
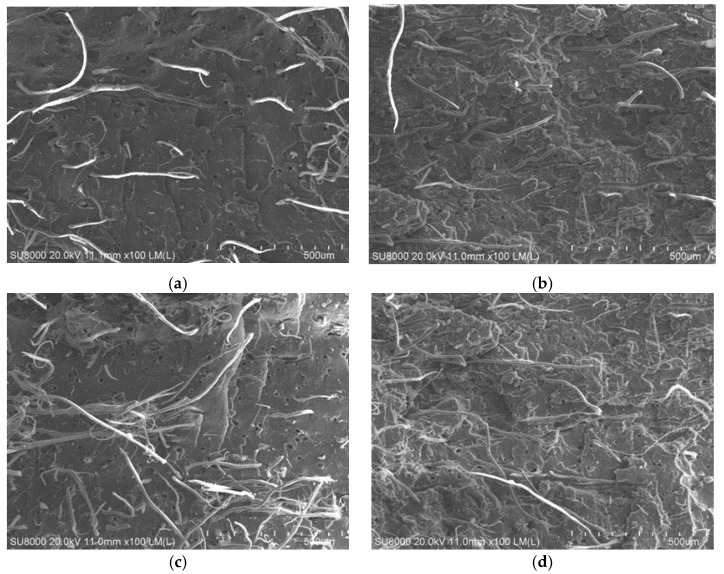
Sectional view of short fiber reinforced rubber composite under SEM (100): (**a**) Y-1; (**b**) Z-1; (**c**) Y-3; (**d**) Z-3.

Subject to different processes, a section of short fiber reinforced rubber composites was observed by scanning electron microscopy after it was broken. A cross-sectional view is shown in Figure 10 with 100 times amplification.

It can be seen from Figure 10 that the orientation and dispersibility of the short fibers were became deteriorated as the screw speed increases. When the screw speed is increased to 30 rpm the degree of orientation of the short fibers in Y-3 was significantly lowered, and the dispersion effect was deteriorated. The short fiber can maintain a certain degree of orientation and dispersibility with the increase of the screw speed when it was extruded by the gear pump. This indicates that using the gear pump can improve the extrusion efficiency while ensuring the quality of the extrusion compound. This is consistent with the experimental results in the performance test.

## 4. Conclusions

The gear pump plays an important role in the processing and performance of short fiber rubber composites. The gear pump used in the processing of the composites could achieve good extrusion characteristics of the extruder at a high rotation speed and improve the compactness and extrusion stability of the rubber compound. Simultaneously, the performance of the composites has been improved in terms of mechanical properties and dynamic mechanical properties.

## Figures and Tables

**Table 1:** Compound formulation.

Component	Formulation A (phr)	Formulation B (phr)
NR	100	100
Carbon black N330	37.4	37.4
Silica	15	15
ZnO	3.6	3.6
Oil	2	2
Anti-aging agent	2	2
Stearic acid	2	2
Accelerator	1.5	1.5
Sulphur	1	1
Coupling agent Si-69	2	2
Resin	2	0
Adhesive	1	0
Aramid short fibers	3	0

**Table 2:** Composite material mixing process.

	First Stage	Second Stage
Mixing parameters	Degree of fill is 65%; chamber wall, rotor and lower top bolt temperature is 40 °C; rotor speed is 40 rpm; Top plug pressure is 0.6 MPa	Roller temperature is (50 ± 5) °C
Mixing processing ^1^	(1) The NR and (Short Aramid Fiber) were incorporated on the internal mixer. (2) After 30 s, ZnO, silica, Si-69, SA, 4010, (SL3020) and half of CB were added into internal mixing. (3) After 30 s, the other half of CB were added into mixer. (4) The Oil was added into mixer when the temperature of mixing achieved 110 °C. (5) The rubber composite (called Master rubber) was discharged when the temperature of mixing achieve 155 °C	The master rubber was added into two-roll mill, The S, NS and (RA 65) were added when the master rubber wrapped on a roller.

^1^ Aramid short fibers, adhesive and resin only incorporated in formulation A.

**Table 3:** Extrusion experimental design.

Numbering	Gear Pump ^1^	Formulation	Screw Speed/rpm
X-1	N	B	10
X-2	N	B	20
X-3	N	B	30
Y-1	N	A	10
Y-2	N	A	20
Y-3	N	A	30
Z-1	Y	A	10
Z-2	Y	A	20
Z-3	Y	A	30

^1^ N means not applicable Gear pump; Y means applicable Gear pump.

**Table 4:** The pressure and temperature value of the mixing rubber at the extruder and extruder head.

Numbering	Maximum of Extruder Pressure/MPa	Maximum of Extruder Temperature/°C	The Pressure of the Extrusion Head/MPa	Extrusion Temperature of the Extrusion Head/°C
X-1	1.89	73	2.88	83
X-2	2.47	77	3.59	94
X-3	3.4	80	4.62	98
Y-1	2.09	75	3.17	85
Y-2	3.17	79	4.18	97
Y-3	3.98	82	5.43	103
Z-1	1.06	74	3.36	84
Z-2	1.64	78	5.08	94
Z-3	2.00	80	6.68	99

**Table 5:** Vulcanization characteristics of extruded products.

Numbering	Mooney Viscosity Pa·s	ML/(dN·m)	MH/(dN·m)	t10/min	t90/min
X-1	38.55	2.44	16.54	4.52	13.63
X-2	36.18	2.3	16.59	4.27	13.32
X-3	35.91	2.26	16.63	4.12	12.53
Y-1	44.51	2.79	17.73	5.47	16.25
Y-2	43.17	2.68	17.86	5.42	15.78
Y-3	44.36	2.82	17.69	5.57	16.97
Z-1	44.65	2.8	17.73	5.43	16.27
Z-2	43.57	2.71	17.82	5.27	15.68
Z-3	42.78	2.55	17.84	5.13	15.20

**Table 6:** Physical and mechanical properties of extruded products.

Numbering	Hardness/°	Tensile Strength/MPa	Tear Strength/KN·m^−^^1^	Wear/μm^3^
X-1	61	18.16	58.65	114
X-2	61.5	18.52	60.75	113
X-3	61.5	18.72	63.05	110
Y-1	67.5	20.85	68.65	102
Y-2	68	21.2	70.12	100
Y-3	67.5	17.41	67.05	120
Z-1	68	21.11	70.81	100
Z-2	68	21.58	71.47	99
Z-3	68	21.65	71.48	98

## References

[1] Hintze C., Boldt R., Wiessner S., Heinrich G. (2013). Influence of processing on morphology in short aramid fiber reinforced elastomer compounds. J. Appl. Polym. Sci..

[2] Yu X.M., Gu B.Q., Zhang B. (2015). Effects of the short-fiber tip geometry and interphase properties on the interfacial debonding behavior of rubber matrix composites. J. Appl. Polym. Sci..

[3] Yu X.M., Gu B.Q., Zhang B. (2015). Effects of short fiber tip geometry and inhomogeneous interphase on the stress distribution of rubber matrix sealing composites. J. Appl. Polym. Sci..

[4] Kumar Sanat K., Benicewicz Brian C., Vaia Richard A., Winey Karen I. (2017). 50th Anniversary Perspective: Are Polymer Nanocomposites Practical for Applications?. Macromolecules.

[5] Gao G.X., Zhang Z.C., Zheng Y.S., Jin Z.H. (2010). Effect of Fiber Orientation Angle on Thermal Degradation and Ablative Properties of Short-Fiber Reinforced EPDM/NBR Rubber Composites. Polym. Compos..

[6] Li Z., Wan J.J., Li Y.Z., Li Y., Zhao F., Zhao S.G. (2019). Effects of coupling agents on the properties of an NR/SBR matrix and its adhesion to continuous basalt fiber cords. J. Appl. Polym. Sci..

[7] Kong H.J., Ding H.Q., Yu M.H., Ding X.M., Qiao M.M. (2019). Influence of poly(p-phenyleneterephalamide) pulp by surface modification with dopamine to nitrile butadiene rubber. Polym. Compos..

[8] Bokobza L. (2019). Natural Rubber Nanocomposites: A Review. Nanomaterials.

[9] Jawaid M., Abdul Khalil H.P.S. (2011). Cellulosic/synthetic fibre reinforced polymer hybrid composites: A review. Carbohydr. Polym..

[10] Jin Z., Luo Z., Yang S.R., Lu S.J. (2015). Influence of complexing treatment and epoxy resin coating on the properties of aramid fiber reinforced natural rubber. J. Appl. Polym. Sci..

[11] Zhang B., Gu B.Q., Yu X.M. (2015). Failure behavior of resorcinol-formaldehyde latex coated aramid short-fiber-reinforced rubber sealing under transverse tension. J. Appl. Polym. Sci..

[12] Luo Z., Chen W.L., Jin Z., Dong F.P., Yang L., Zheng Q. (2018). Epoxy resin modified maleic anhydride-grafted-liquid polybutadiene on the properties of short aramid fiber reinforced natural rubber composite. Polym. Compos..

[13] Miedzianowska J., Maslowski M., Strzelec K. (2019). Thermoplastic Elastomer Biocomposites Filled with Cereal Straw Fibers Obtained with Different Processing Methods-Preparation and Properties. Polymers.

[14] Summerscales J., Green D., Guild F.J. (1993). Effect of processing dwell-time on the microstructure of a fibre-reinforced composite. J. Microsc..

[15] Kashani M.R. (2009). Aramid-short-fiber reinforced rubber as a tire tread composite. J. Appl. Polym. Sci..

[16] Ozkoc G., Bayram G., Bayramli E. (2005). Short glass fiber reinforced ABS and ABS/PA6 composites: Processing and characterization. Polym. Compos..

[17] Thomas R.P.K.S. (1995). Tear and Processing Behaviour of Short Sisal Fibre Reinforced Styrene Butadiene Rubber Composites. Polym. Int..

[18] Zhang B., Gu B., Yu X. (2014). Failure behavior of resorcinol-formaldehyde latex coated aramid short-fiber-reinforced rubber sealing under transverse tension. J. Appl. Polym. Sci..

[19] Lekube B., Purgleitner B., Renner K., Burgstaller C. (2019). Influence of Screw Configuration and Processing Temperature on the Properties of Short Glass Fiber Reinforced Polypropylene Composites. Polym. Eng. Sci..

[20] Wang C.S., Liu C.J., Bian H.G. (2009). Radial orientation mechanism and experimental research of short fiber in tread compound. J. Donghua Univ..

[21] Wang C.S., Zhang D.W., Li L. (2014). Experimental study of integration and polyblends performance for improved mixing-extruding machine. J. Donghua Univ..

[22] Li L., Wang C.S., Zhang D.W. (2014). Effects of radial-orientation-die structure parameters on properties of short fiber and rubber composite material. J. Donghua Univ. (Engl. Ed.).

[23] Shirazi M., Talma A.G., Noordermeer J.W.M. (2013). Viscoelastic properties of short aramid fibers-reinforced rubbers. J. Appl. Polym. Sci..

[24] Hotaka T., Ishikawa Y., Mori K. (2005). Effect of Compound Ingredients on Adhesion between Rubber and Brass-Plated Steel Cord. Rubber Chem. Technol..

[25] Patil P. (2005). Mechanistic Investigation of Rubber-Brass Adhesion: Effect of Formulation Ingredients. Ph.D. Thesis.

[26] Kargarzadeh H., Mariano M., Huang J., Lin N., Ahmad I., Dufresne A., Thomas S. (2017). Recent developments on nanocellulose reinforced polymer nanocomposites: A review. Polymer.

[27] Saba N., Jawaid M., Alothman Othman Y., Paridah M.T. (2016). A review on dynamic mechanical properties of natural fibre reinforced polymer composites. Constr. Build. Mater..

[28] Thaptong P., Sae-Oui P., Sirisinha C. (2016). Effects of silanization temperature and silica type onproperties of silica-filled solution styrene butadiene rubber (SSBR) for passenger car tire treadcompounds. J. Appl. Polym. Sci..

[29] Gabriel C.F.S., de Alencar Padua Gabino A., de Sousa A.M.F., Furtado C.R.G., Nunes R.C.R. (2019). Tire tread rubber compounds with ternary system filler based on carbon black, silica, and metakaolin: Contribution of silica/metakaolin content on the final properties. J. Elastomers Plast..

